# A global evaluation of advanced dosimetry in transarterial radioembolization of hepatocellular carcinoma with Yttrium-90: the TARGET study

**DOI:** 10.1007/s00259-022-05774-0

**Published:** 2022-04-08

**Authors:** Marnix Lam, Etienne Garin, Marco Maccauro, S. Cheenu Kappadath, Daniel Y. Sze, Cuneyt Turkmen, Murat Cantasdemir, Paul Haste, Ken Herrmann, Hamad Saleh Alsuhaibani, Matthew Dreher, Kirk D. Fowers, Riad Salem

**Affiliations:** 1 Department of Radiology and Nuclear Medicine, University Medical Center Utrecht, Huispostnummer E01.132, Postbus 85500, 3508 GA Utrecht, The Netherlands; 2 Nuclear Medicine Department, Eugene Marquis Center, Rennes, France; 3 Nuclear Medicine Unit, Fondazione IRCCS Istituto Nazionale Dei Tumori, Milan, Italy; 4 Department of Imaging Physics, University of Texas MD Anderson Cancer Center, Houston, TX USA; 5 Division of Interventional Radiology, Stanford University, Stanford, CA USA; 6 Department of Nuclear Medicine, Istanbul Faculty of Medicine, Istanbul University, Istanbul, Turkey; 7 Division of Interventional Radiology, Istanbul Florence Nightingale Hospital, Istanbul, Turkey; 8 Department of Clinical Radiology and Imaging Sciences, Indiana University School of Medicine, Indianapolis, IN USA; 9 Department of Nuclear Medicine, University of Duisburg-Essen, and German Cancer Consortium (DKTK)-University Hospital Essen, Essen, Germany; 10 Department of Radiology, King Faisal Specialist Hospital, Riyadh, Saudi Arabia; 11 Boston Scientific Corporation, Marlborough, MA USA; 12 Department of Radiology, Northwestern Feinberg School of Medicine, Chicago, IL USA

**Keywords:** Radioembolization, Yttrium-90, Dosimetry, Hepatocellular carcinoma

## Abstract

**Purpose:**

To investigate the relationships between tumor absorbed dose (TAD) or normal tissue absorbed dose (NTAD) and clinical outcomes in hepatocellular carcinoma (HCC) treated with yttrium-90 glass microspheres.

**Methods:**

TARGET was a retrospective investigation in 13 centers across eight countries. Key inclusion criteria: liver-dominant HCC with or without portal vein thrombosis, < 10 tumors per lobe (at least one ≥ 3 cm), Child–Pugh stage A/B7, BCLC stages A–C, and no prior intra-arterial treatment. Multi-compartment pre-treatment dosimetry was performed retrospectively. Primary endpoint was the relationship between ≥ grade 3 hyperbilirubinemia (such that > 15% of patients experienced an event) without disease progression and NTAD. Secondary endpoints included relationships between (1) objective response (OR) and TAD, (2) overall survival (OS) and TAD, and (3) alpha fetoprotein (AFP) and TAD.

**Results:**

No relationship was found between NTAD and ≥ grade 3 hyperbilirubinemia, which occurred in 4.8% of the 209 patients. The mRECIST OR rate over all lesions was 61.7%; for the target (largest) lesion, 70.8%. Responders and non-responders had geometric mean total perfused TADs of 225.5 Gy and 188.3 Gy (*p* = 0.048). Probability of OR was higher with increasing TAD (*p* = 0.044). Higher TAD was associated with longer OS (HR per 100 Gy increase = 0.83, 95% CI: 0.71–0.95; *p* = 0.009). Increased TAD was associated with higher probability of AFP response (*p* = 0.046 for baseline AFP ≥ 200 ng/mL).

**Conclusion:**

Real-world data confirmed a significant association between TAD and OR, TAD and OS, and TAD and AFP response. No association was found between ≥ grade 3 hyperbilirubinemia and NTAD.

**Trial registration number:**

NCT03295006.

**Supplementary Information:**

The online version contains supplementary material available at 10.1007/s00259-022-05774-0.

## Introduction

Over the past decade, the management of disease in patients with hepatocellular carcinoma (HCC) has evolved to include a wide variety of therapeutic options, including systemic and locoregional techniques. A well-established locoregional treatment option is transarterial radioembolization (TARE) using yttrium-90 (^90^Y) glass microspheres (TheraSphere™, Boston Scientific Corporation, Marlborough, MA, USA). Several studies have demonstrated that TARE provides excellent clinical outcomes, including an improvement in survival [1, 2]. Improved results are achieved when the tumor absorbed dose (TAD) is increased without worsening the safety profile [1, 3, 4].

Historically, dosimetry recommendations were to target an average absorbed dose of 120 Gy to the perfused liver for lobar treatment, or > 190 Gy to the tumor for ablative radiation in ≤ 2 Couinaud liver segments, i.e., radiation segmentectomy [5, 6]. Using these recommendations, no distinction was made between TAD and normal tissue absorbed dose (NTAD). Recently, however, prospective and retrospective studies have demonstrated improved outcomes based on personalized dosimetry techniques utilized in both single and multi-compartment dosimetry. In single compartment dosimetry, a pathological response is achieved in the vast majority of patients if ablative radiation is delivered to a limited perfused volume with an absorbed dose exceeding 400 Gy [2, 7, 8]. Lobar infusion, however, encompasses a larger liver volume, requiring a balance between the achievable TAD and evaluation of the NTAD to minimize normal tissue toxicity [9, 10]. Multi-compartment dosimetry involves the recognition that tumor and normal liver tissue have distinctly different levels of microsphere accumulation with important consequences with regard to efficacy and toxicity. Pre-treatment technetium-99 m macroaggregated albumin (^99m^Tc-MAA) images are used for predictive dosimetry, while post-treatment ^90^Y images can be used for confirmatory dosimetry [1, 9]. An appropriate catheter position and delivery technique are required to achieve reproducibility between pre-treatment ^99m^Tc-MAA and post-treatment ^90^Y SPECT/CT bremsstrahlung or PET/CT distribution images [4, 11–14]. Benefits of multi-compartment dosimetry include more predictable TADs administered to achieve ablative results, improved tumor response and portal vein tumor-thrombosis (PVT) response with ^99m^Tc-MAA predicted targeting, and predictability of the atrophy/hypertrophy complex (AHC) in borderline resectable patients [1, 9, 15, 16]. Recent studies have highlighted the evolution of recommended dose ranges for both single compartment and multi-compartment dosimetry, particularly for patients for whom the goal of treatment is curative by means of tissue ablation or subsequent surgical resection, but also in a palliative setting by improving tumor and PVT response and increasing overall survival (OS). While a number of single center studies have highlighted improved outcomes by increasing the TAD, collection of real-world data is needed to evaluate a broad range of TAD, NTAD, and practice patterns to determine the relationship with clinical and safety outcomes in patients receiving ^90^Y glass microspheres.

Here, we present the primary results of the TARGET clinical evaluation study, specifically, the primary aims of determining relationships between TAD or NTAD and treatment-related adverse events (AEs), treatment response, and OS.

## Methods

### Study design

The TheraSphere™ Advanced Dosimetry Retrospective Global Study Evaluation in Hepatocellular Carcinoma Treatment (TARGET) study was an international, multi-center, retrospective, single-arm study of patients from 13 centers located across eight countries who were treated using ^90^Y glass microspheres for HCC. Patients included were treated between January 1^st^, 2010 and December 31^st^, 2017. Sites included University Medical Center Utrecht, Netherlands; Northwestern University, Chicago, IL, USA; Eugene Marquis Center, Rennes, France; Indiana University School of Medicine, Indianapolis, IN, USA; MD Anderson Cancer Center, Houston, TX, USA; Stanford University, Stanford, CA, USA; University of Florida College of Medicine, Gainesville, FL, USA; Centre Hospitalier, Universitaire Vaudois, Lausanne, Switzerland; Universitatsklinikum, Essen, Germany; Foundation IRCCS Istituto Nazionale Tumori, Milan, Italy; Istanbul University Medical School, Istanbul, Turkey; Florence Nightingale Hastanesi, Istanbul, Turkey; King Faisal Specialist Hospital and Research Centre, Saudi Arabia. Protocols were approved by each site’s respective Institutional Review Boards (IRBs) and/or Independent Ethics Committees (IECs).

Patients were treated according to instructions for use of the device and local procedure. They were selected in consecutive reverse chronological order at each site to minimize patient selection bias.

The TARGET study consisted of three sub-studies: one evaluating the inter-site variability of pre-treatment dosimetry; one evaluating dosimetry software/methodology accuracy and reproducibility; and the primary study to collect clinical data to generate predictive models for NTAD and TAD association with clinical outcomes.

### Inclusion/exclusion criteria

In addition to the standard inclusion criteria for patients undergoing TARE, the key patient inclusion criteria in this retrospective analysis were liver-dominant disease; up to 10 well-defined HCC tumor(s), with at least one tumor ≥ 3 cm, with or without PVT; Child–Pugh stage A or B7; BCLC stage A, B, or C; male or female; aged 18 years or older; bilirubin ≤ 2 mg/dL; and tumor replacement < 50% of total liver volume by diagnostic imaging. Patients were required to have had diagnostic imaging consisting of multi-phase contrast enhanced CT or contrast enhanced MRI within 3 months prior to ^99m^Tc-MAA SPECT or SPECT/CT imaging; received an infusion of ^99m^Tc-MAA and ^90^Y glass microspheres from a single location sufficient to cover the tumor(s) based on angiography; had clinical evaluation and laboratory evaluation (at least bilirubin) at baseline. Accumulation of ^99m^Tc-MAA within PVT was not an inclusion criterion.

Exclusion criteria included prior external beam radiation treatment to the liver; prior loco-regional liver-directed therapy (e.g., transarterial chemoembolization [TACE] and/or TARE); prior liver resection or transplantation; any anti-cancer therapy between first TARE treatment and 3-month imaging; hepatic vein invasion; and administration to ≤ 2 segments (i.e., radiation segmentectomy). Radiation segmentectomy patients were excluded as ablative radiation to a minimal liver volume would skew the results.

### Data collection

Patient data were collected through a retrospective review of patient records to identify demographic characteristics, (disease-specific) medical history, Child–Pugh and Barcelona Clinic Liver Cancer (BCLC) status, Eastern Cooperative Oncology Group (ECOG) performance status, treatment-specific variables, and potentially dose-related adverse events (AEs). Demographic data collected included age, gender, race, and ethnicity. Medical history was deemed clinically significant (potentially relevant to identified AEs) by the site investigator. All potentially relevant ongoing medical conditions and baseline symptoms arise from treatment of those conditions to be a part of the patient’s medical history. Additionally, medical histories included clinical laboratory results (biochemistry, coagulation, hematology, and tumor marker) and concomitant medication. Disease-specific medical history included date of HCC diagnosis and presence of liver disease (etiology), hepatic encephalopathy, ascites, and subsequent treatments following diagnosis. Treatment-specific variables included those related to the preparation and imaging of ^99m^Tc-MAA, the administration of ^99m^Tc-MAA and ^90^Y glass microspheres, and specific treatment parameters. Clinical evaluation results were collected at baseline (+ / − 30 days), 90 days (+ / − 30 days), and 180 days (+ / − 30 days) after treatment. Laboratory testing results were collected at baseline (+ / − 30 days), 42 days (+ / − 17 days), and 90 days (+ / − 30 days) after treatment. Imaging data were collected at baseline (+ / − 30 days), 42 days (+ / − 17 days), 90 days (+ / − 30 days), 180 days (+ / − 30 days) after treatment; any additional imaging data available between 180 and 400 days was also collected. AEs were collected up to 90 days and were defined using the National Cancer Institute’s Common Terminology for Adverse Events (CTCAE) version 4.02.

### Primary and secondary endpoints

The primary endpoint was to determine the relationship between total perfused NTAD and occurrence of ≥ grade 3 hyperbilirubinemia without disease progression in 15% or more of patients treated. This endpoint was selected based on consensus between study investigators during a study planning meeting. Secondary endpoints included assessment of treatment-related (S)AEs within 90 days of treatment with ^90^Y glass microspheres and their relationship with NTAD; determining the relationship between predicted TAD and best objective response (defined as complete response [CR] or partial response [PR] by mRECIST and RECIST 1.1); OS; the relationship between TAD and OS; and the relationship between tumor marker (alpha fetoprotein [AFP]) response and TAD. AEs and SAEs related or potentially related to TARE were collected up to 90 days after treatment. The maximum grade AE was recorded during this window. Best response was evaluated based on available imaging between 25 and 400 days post treatment, e.g., evaluable target lesions available at Day 90 (*n* = 130) and Day 180 (*n* = 74).

### Imaging and dosimetry

Imaging was based upon institutional practice but required, at a minimum, angiography documenting the catheter position for ^99m^Tc-MAA and ^90^Y glass microsphere administration, diagnostic contrast enhanced imaging (CT or MRI), and ^99m^Tc-MAA SPECT or SPECT/CT imaging. Type of diagnostic and response imaging used at each site (e.g., CT, MRI) was recorded.

Dosimetry analyses were performed by local clinical teams as determined using standard instructions for dosimetry calculations. Lung shunt fraction (LSF) was calculated on planar ^99m^Tc-MAA images per routine clinical practice (maximum 30 Gy for single administration; cumulative 50 Gy for multiple administrations).

Multi-compartment dosimetry was calculated retrospectively using Simplicit^90^Y™ software (Mirada Medical LTD., Oxford, UK). Dosimetry analysis included three main steps: registration, segmentation, and dose calculation. For the ^99m^Tc-MAA images, a co-registered SPECT/CT was utilized over a SPECT, when available (178 SPECT/CT and 31 SPECT).

Registration was performed using data from diagnostic contrast enhanced CT/MRI with ^99m^Tc-MAA SPECT/CT or SPECT. The registration was adjusted as needed using dosimetry software tools, then the quality of the registration was evaluated. The volume of interest (VOI) segmentation process involved the following: (i) segmenting lung volume on planar ^99m^Tc-MAA scintigraphy; and (ii) segmenting whole liver, whole liver normal tissue, perfused liver, perfused normal liver (i.e., perfused liver minus sum of all tumors ≥ 2 cm), total perfused tumors (i.e., sum of all tumors ≥ 3 cm) and target lesion (single largest lesion) on multiphase contrast enhanced CT or MRI. Tumors ≤ 2 cm were conservatively added to normal perfused liver because of increased dosimetry errors at low volumes caused by registration errors, limited SPECT resolution, and partial volume effects. For accurate dosimetry, the total perfused tumor volume included all tumors ≥ 3 cm. A board-certified radiologist, not involved in assessment of tumor response, inspected all segmented volumes to confirm that the volume corresponded to the correct description and evaluated the quality of the registration. Dose calculation was performed using the VOI and counts from the registered ^99m^Tc-MAA SPECT/CT according to the MIRD schema using a patient relative calibration factor and the local deposition method. The mean total perfused tumor absorbed dose was calculated based on a tumor size weighted average. NTAD and TAD were calculated primarily using ^99m^Tc-MAA imaging; ^90^Y PET was only available for a minority of patients.

When the imaging described above was not available, alternative modalities were used, including SPECT being used instead of SPECT/CT, and, in one patient, cone beam CT (CBCT) was used instead of CT for tumor volume assessment.

### Statistics

The total projected sample size of between 200 and 300 patients was determined using simulated logistic regression curves of the relationship between the occurrence of ≥ grade 3 hyperbilirubinemia and NTAD, where the width of the 95% confidence intervals for the simulated curves suggested that between 200 and 300 patients would provide a reliable estimate of the adverse event of interest.

The main analysis population included all patients enrolled in the study who satisfied the eligibility criteria. Logistic regression was used to determine the relationship between the occurrence of ≥ grade 3 hyperbilirubinemia and NTAD, the relationship between OR and TAD, and between AFP response and TAD. Kaplan–Meier methodology was used to analyze OS. Cox regression was used to evaluate the relationship between TAD and OS.

Logistic regression was also used to assess the relationship between OR and TAD after taking account of pre-defined covariates of interest, according to the following steps:

- Step 1: Each covariate was included, one at a time, together with TAD, in a series of univariable models. If the covariate had 2-sided *p* value < 0.1, it was included in the model selection procedure described in Step 2.
- Step 2: A backward elimination procedure was used to determine the final multivariable model, starting with all the significant covariates identified in Step 1. A 2-sided significance level of 10% was used for a covariate to remain in the model. The process was repeated until all the remaining covariates had 2-sided *p* values < 0.1.

Similarly, Cox regression was used to assess the relationship between OS and TAD after taking account of pre-defined covariates of interest, according to the steps described above.

## Results

### Patient demographics and baseline characteristics

According to the study protocol, patients treated with ^90^Y glass microspheres between 1st January 2010 and 31st December 2017 were screened for inclusion. A total of 209 met the inclusion criteria and were included in the study. Baseline patient characteristics are included in Table 1. Median follow-up after treatment was 13.3 months (range: 0.6, 98.0). Of those classified as BCLC C (*n* = 114), 69 had PVT, and 45 were ECOG ≥ 1 without PVT; of the 69 with PVT, 42 were ECOG 0.

**Table 1:** Baseline characteristics

Patient characteristics	Treated population (*N* = 209) *N* (%)
Median age (range), years	66 (27–87)
Gender, male	166 (79.4%)
Etiology*	
Hepatitis B	31 (14.8%)
Hepatitis C	69 (33.0%)
Alcohol	48 (23.0%)
Non-alcoholic steatohepatitis	20 (9.6%)
Liver disease of unknown etiology	32 (15.3%)
Other	28 (13.4%)
Cirrhosis	185 (88.5%)
ECOG status	
0	135 (64.6%)
1	67 (32.1%)
≥ 2	7 (3.4%)
BCLC status	
A	27 (12.9%)
B	68 (32.5%)
C	114 (54.5%)
Child–Pugh status	
A (5–6)	187 (89.5%)
B7	22 (10.5%)
Unilobar or bilobar disease	
Unilobar	148 (70.8%)
Bilobar	61 (29.2%)
With PVT	69 (33.0%)
Location of target lesion	
Left lobe	30 (14.4%)
Right lobe	179 (85.6%)
Target lesion longest diameter (RECIST 1.1)	
≥ 3 to < 5 cm	41 (19.6%)
≥ 5 to < 8 cm	72 (34.4%)
≥ 8 cm	96 (45.9%)
Total number of lesions (target and non-target)	
1	145 (69.4%)
2	45 (21.5%)
3	14 (6.7%)
4–10	5 (2.4%)
Prior Sorafenib treatment	21 (10.0%)
Time from HCC diagnosis to TARE treatment (months)	
Mean (SD)	3.7 (8.9)
Median (range)	2.1 (0.2–79.3)

^*^Patients could have multiple etiologies

### Dosimetry

Median administered activity was 2.7 GBq (range: 0.8, 9.3; Table 2) in a median perfused volume of 1107.3 mL (range: 250.4, 3389.7; Table 2). The median total perfused liver fraction was 63.9% (range: 14.9, 97.0), with a median whole liver volume of 1806.3 mL (range: 667.2, 4283.5). The median whole liver NTAD was 48.1 Gy (range: 5.4, 166.0; Table 2). Treatment with ^90^Y glass microspheres resulted in a median total perfused liver absorbed dose of 117.3 Gy (range: 27.1, 286.1). Median total perfused TAD was 216.0 Gy (range: 14.0, 1130.4) over a median total perfused tumor volume of 204.8 mL (range: 7.9, 1688.5). Median total perfused NTAD was 87.3 Gy (range: 16.8, 270.1) over a median total perfused non-tumorous volume of 838.0 cm^3^ (range: 109.0, 2495.7). Median lung shunt fraction was 4.0% (range: 0, 31.0); median anticipated lung absorbed dose was 6.6 Gy (range: 0.2, 43.1).

**Table 2:** Summary of volumetry and dosimetry data

	Treated population (*N* = 209)
Activity administered (GBq)	
Mean (SD)	2.9 (1.3)
Median (range)	2.7 (0.8–9.3)
Treatment administration week after calibration	
First week (*n* [%])	116 (60.7)
Second week (*n* [%])	75 (39.3)
Lung shunt fraction (%)	
Mean (SD)	5.8 (6.3)
Median (range)	4.0 (0–31.0)
Whole liver volume (mL)	
Mean (SD)	1933.7 (652.5)
Median (range)	1806.3 (667.2–4283.5)
Whole liver normal tissue volume (mL)	
Mean (SD)	1626.5 (554.6)
Median (range)	1514.2 (627.9–3600.0)
Whole liver normal tissue absorbed dose (Gy)	
Mean (SD)	48.9 (24.5)
Median (range)	48.1 (5.4–166.0)
Total perfused liver volume (mL)	
Mean (SD)	1178.8 (519.5)
Median (range)	1107.3 (250.4–3389.7)
Total perfused liver (%)	
Mean (SD)	61.1 (16.1)
Median (range)	63.9 (14.9–97.0)
Total perfused liver absorbed dose (Gy)	
Mean (SD)	122.4 (43.7)
Median (range)	117.3 (27.1–286.1)
Total perfused tumor volume (mL)	
Mean (SD)	307.1 (322.7)
Median (range)	204.8 (7.9–1932.8)
Total perfused tumor absorbed dose (TAD; Gy)	
Mean (SD)	254.5 (166.4)
Median (range)	216.0 (14.0–1130.4)
Target lesion volume (mL)	
Mean (SD)	299.3 (330.0)
Median (range)	198.3 (7.9–1932.8)
Target lesion absorbed dose (Gy)	
Mean (SD)	257.6 (171.7)
Median (range)	217.1 (14.0–1130.4)
Total perfused normal tissue volume (mL)	
Mean (SD)	873.6 (429.5)
Median (range)	838.0 (109.0–2495.7)
Total perfused normal tissue absorbed dose (NTAD; Gy)	
Mean (SD)	92.6 (42.9)
Median (range)	87.3 (16.8–270.1)

### Primary endpoint

No relationship was found between NTAD and occurrence of ≥ grade 3 hyperbilirubinemia without disease progression (*p* = 0.6 by logistic regression). This was likely due to the fact that hyperbilirubinemia occurred in only 4.8% of patients (including one case of grade 4 liver failure with missing bilirubin measurement) and therefore did not meet the threshold of 15% of patients set forth in the initial primary endpoint definition. The range of NTADs was well tolerated, with no unexpected safety signals generated. Additionally, logistic regression analyses performed to assess the relationship between NTAD and all dose-related AEs (defined as any grade of hyperbilirubinemia, ascites, pain, fatigue, nausea, or post-embolization syndrome) showed no evidence of an association between increased NTAD and the occurrence of these dose-related AEs.

### Secondary endpoints

**Fig. 1:**
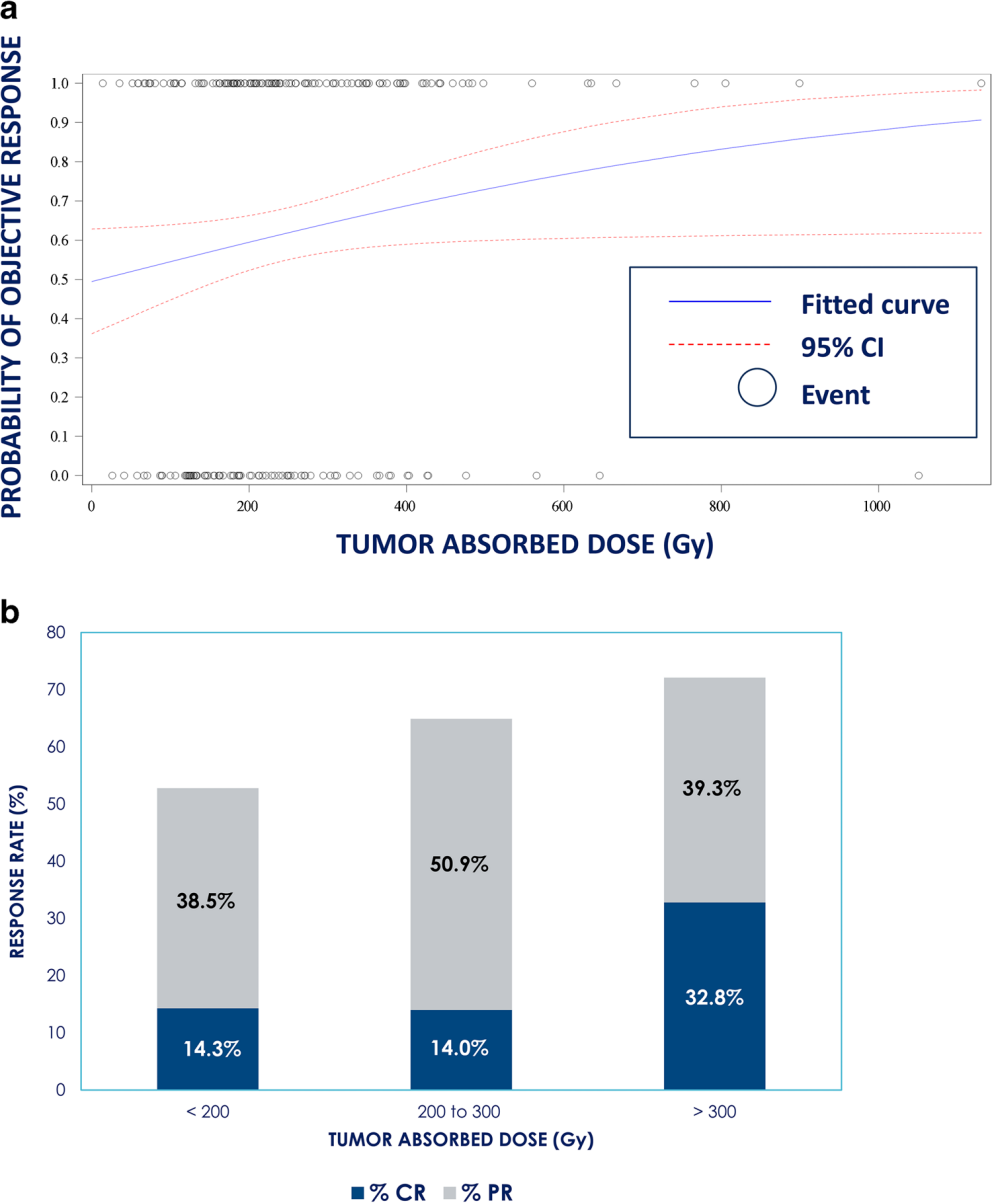
Objective response by mRECIST and tumor absorbed dose (TAD). **a**) Logistic regression curve of probability of objective response by TAD. **b**) Objective response rate (best response during follow-up) by TAD subgroups

Objective response rate (ORR), defined as complete or partial response by mRECIST, was 61.7% (129/209; 95% confidence interval [CI] = 55.0–68.0%) over all lesions; by RECIST 1.1, ORR was 34.4% (72/209; 95% CI = 28.3–41.1%). The median target lesion absorbed dose was 217.1 Gy (range: 14.0–1130.4 Gy); the median total perfused TAD was 216.0 Gy (range: 14.0–1130.4 Gy; Table 2). Logistic regression demonstrated that increasing total perfused TAD was associated with higher ORR for both mRECIST and RECIST 1.1 measurements (*p* = 0.044 and *p* = 0.030, respectively; Fig. 1a). ORR in the target lesion by mRECIST was 70.8% (148/209; 95% CI = 64.3–76.6%). The rate of patients with CR increased from 14.3% for TAD < 200 Gy to 32.8% for TAD > 300 Gy. ORR in the target lesion by RECIST 1.1 was 38.8% (81/209; 95% CI = 32.4–45.5%). By mRECIST, the 129 responders had a significantly higher geometric mean total perfused TAD (225.5 Gy; 95% CI = 201.0–253.0 Gy) compared with the 80 non-responders (188.3 Gy; 95% CI = 164.6–215.3 Gy; *p* = 0.048 by 2-sample *t* test). This significant difference held for RECIST 1.1 for the 72 responders (242.1 Gy; 95% CI = 208.6–280.9 Gy) and the 137 non-responders (195.5 Gy; 95% CI = 175.5–217.8 Gy; *p* = 0.022).

**Fig. 2:**
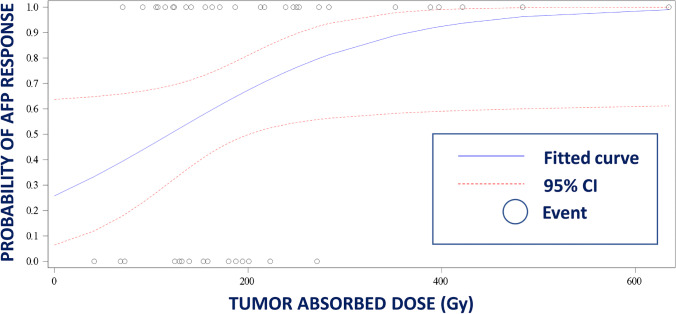
Logistic regression curve of probability of alpha fetoprotein (AFP) response 90 days post-treatment by tumor absorbed dose (TAD) for patients with baseline AFP levels of ≥ 200 ng/mL

Of the 71 patients with AFP ≥ 200 ng/mL at baseline, 38.0% (27/71) had a response (defined as a ≥ 50% decrease from baseline) at 90 days post-treatment; of the 107 patients with AFP ≥ 20 ng/mL at baseline, 38.3% (41/107) experienced a response (defined as a ≥ 20% decrease from baseline) 90 days post-treatment. Figure 2 shows the probability of AFP response 90 days post-treatment by TAD for patients with baseline AFP levels of ≥ 200 ng/mL.

**Fig. 3:**
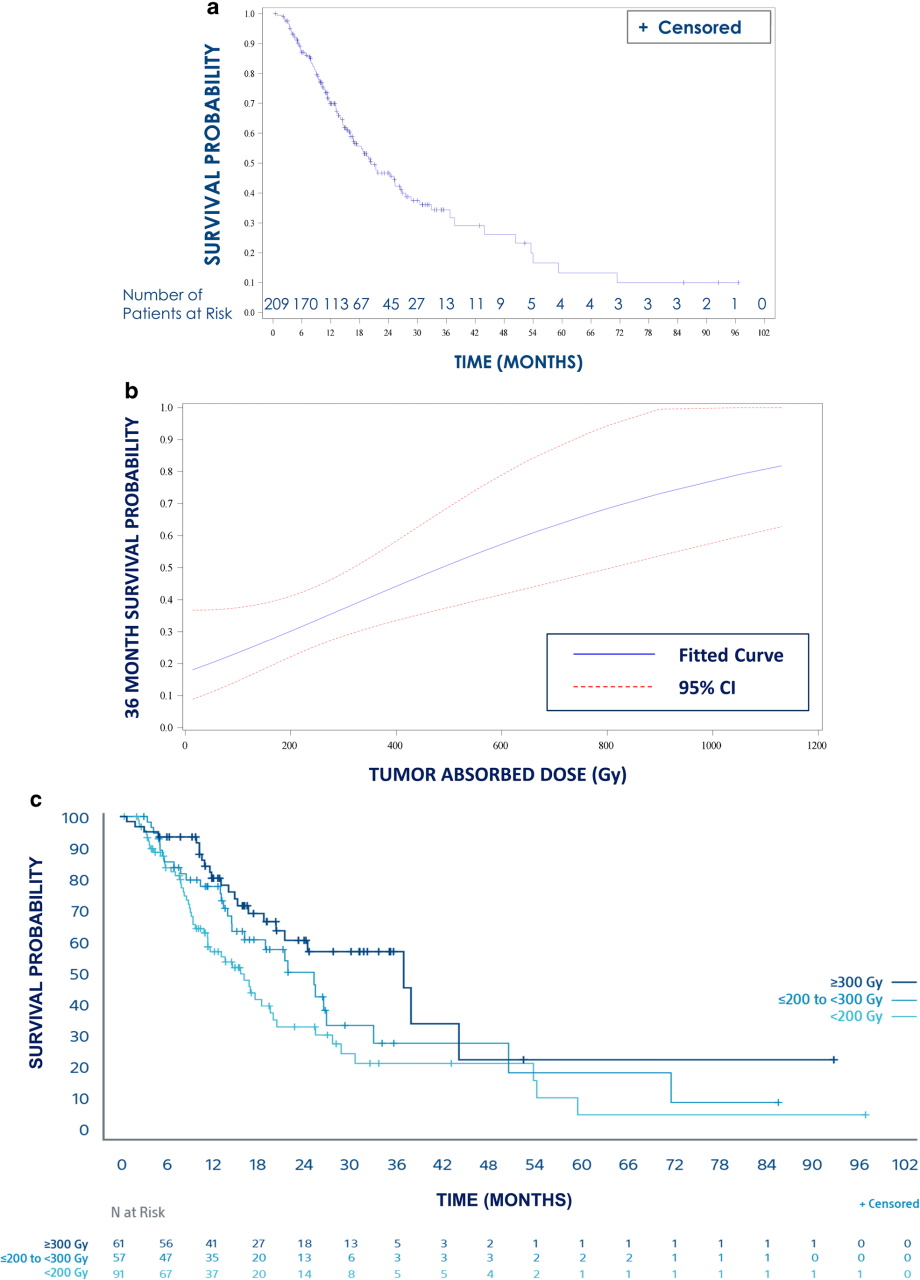
Overall survival. **a** Kaplan–Meier curve for all 209 patients. **b** Cox regression curve of probability of survival at 36 months by tumor absorbed dose TAD). **c** Kaplan–Meier curves by tumor absorbed dose subgroups

Median OS across all patients was 20.3 months (95% CI = 16.7–26.4 months; Fig. 3a). Increased TAD was associated with longer OS by Cox regression (Fig. 3b). The OS hazard ratio, corresponding to every 100 Gy increase in TAD, was 0.826 (95% CI = 0.71–0.95; *p* = 0.009); this translates to a 17% improvement in survival probability on the log scale per 100 Gy increase in TAD.

Additional analyses were conducted for ORR and OS stratifying patients into dose subgroups of < 200 Gy, ≥ 200 Gy and < 300 Gy, and ≥ 300 Gy. ORR by mRECIST over all lesions in patients with a TAD < 200 Gy was 52.7%; with a TAD of 200–300 Gy, 64.9%; and with a TAD > 300 Gy, 72.1% (Fig. 1b). Median OS in patients with a TAD < 200 Gy was 16.1 months (95% CI: 11.3–19.4 months); with a TAD of 200–300 Gy, 25.1 months (95% CI: 14.5–32.9 months); and with a TAD > 300 Gy, 36.7 months (95% CI: 20.2–43.9 months; Fig. 3c). Using Cox regression, a significant difference in OS was found across the 3 dose subgroups (*p* = 0.0052) and specifically between patients who had a TAD < 200 Gy and those with a TAD > 300 Gy (*p* = 0.0014; Fig. 3c).

TAD continued to have a statistically significant (i.e., *p* < 0.05) impact on ORR and OS in the final multivariable analyses after taking account of over 20 additional parameters to TAD, including tumor size, number of lesions, unilobar vs bilobar disease, BCLC stage, ALBI (Albumin-Bilirubin) score, Child–Pugh score, HCC etiology, and presence of PVT (Online Resource 1, Tables S1, S2, and S3).

### Adverse events

Of the 209 patients included, 131 (62.7%) experienced at least one treatment-related adverse event; of these, 43 (20.6%) patients experienced at least one treatment-related adverse event of grade 3 or higher (Table 3). The most frequently observed adverse events included ascites (38/209, 18.2%), fatigue (34/209, 16.3%), elevated bilirubin (29/209, 13.9%), abdominal pain (26/209, 12.4%), and decreased lymphocyte count (25/209, 12.0%). In total, nine cases with grade 3 or higher elevated bilirubin were identified. Fifteen grade 4 related AEs were reported across nine patients; these included three cases of decreased lymphocyte count, and ascites, decreased appetite, edema, “general physical health deterioration,” decreased blood albumin, hepatic failure (this case was conservatively added to the nine cases of grade 3 or higher hyperbilirubinemia for primary endpoint analysis), hepatic cirrhosis, lymphopenia, dyspnoea, encephalopathy, peritonitis, sepsis, one case each.

**Table 3:** Treatment related adverse events experienced by ≥ 5% of patients by CTCAE grade

Adverse event term	CTCAE grade				Total *N* = 209 *n* (%)
	1 *n* (%)	2 *n* (%)	3 *n* (%)	4 *n* (%)	
Patients with adverse events	52 (24.9)	36 (17.2)	34 (16.3)	9 (4.3)	131 (62.7)
Ascites	15 (7.2)	13 (6.2)	9 (4.3)	1 (0.5)	38 (18.2)
Fatigue	22 (10.5)	11 (5.3)	1 (0.5)	0	34 (16.3)
Abdominal pain	18 (8.6)	6 (2.9)	2 (1.0)	0	26 (12.4)
Elevated bilirubin	6 (2.9)	14 (6.7)	9^a^ (4.3)	0	29 (13.9)
Lymphocyte count decreased	3 (1.4)	6 (2.9)	13 (6.2)	3 (1.4)	25 (12.0)
Asthenia	14 (6.7)	4 (1.9)	1 (0.5)	0	19 (9.1)
Decreased appetite	10 (4.8)	2 (1.0)	0	1 (0.5)	13 (6.2)
Nausea	8 (3.8)	3 (1.4)	0	0	11 (5.3)

^a.^An additional patient with an AE of grade 3 hepatic failure was included in the logistic regression analysis as having ≥ grade 3 hyperbilirubinemia without disease progression

## Discussion

A key clinical data gap addressed in the TARGET study was the relationship between TAD and NTAD and clinical outcomes across a large group of real-world patients. This included patients across BCLC stages, with and without PVT, different tumor sizes, and variable tumor hypervascularity. The natural distribution of tumor and normal tissue volumes, perfused volume, and tumor hypervascularity provided a broad range of TAD and NTAD for analysis of associations with clinical outcomes. As a multinational study, the TARGET study generated evidence to provide more insight into selecting appropriate TAD while balancing the NTAD. No association was found between ≥ grade 3 hyperbilirubinemia without disease progression and NTAD, due in part to the fact that the incidence was too low to generate an association (prespecified threshold of 15% or more patients experiencing an event). Hyperbilirubinemia of grade 3 or higher was selected as an appropriate measure of toxicity as it has been used in previous publications [17]. Patients with a tumor response had a significantly higher TAD than those who did not respond; furthermore, the probability of achieving a response was higher with increased TAD. Higher TAD was also found to be associated with increased probability of an AFP response and longer OS.

The dosimetry recommendations for TARE with ^90^Y glass microspheres as a locoregional treatment for HCC have evolved across all stages of HCC [1, 2, 18, 19]. Dosimetry may be performed using either single or multi-compartment dosimetry and may be personalized depending on the treatment intent. Multi-compartment dosimetry involves the evaluation of both TAD and NTAD to achieve a balance in safety (NTAD) and efficacy (TAD) and is particularly beneficial in patients undergoing large liver fraction infusions [1, 17]. Recent publications have highlighted the ability of ^99m^Tc-MAA as a surrogate for ^90^Y glass microsphere distribution in HCC patients when consistent catheter and infusion techniques are utilized [4, 9–12]. Commercial software platforms have been developed to reduce dosimetry analysis time and facilitate improved reproducibility.

TARGET evaluated HCC patients with a median 63.9% perfused liver volume and a median average perfused liver absorbed dose of 117.3 Gy; this represents a historically relevant lobar treatment population. While an association between NTAD and occurrence of ≥ grade 3 hyperbilirubinemia was not identified, recent publications demonstrated that a higher NTAD was well tolerated in patients who had preserved liver function (bilirubin normal) and sufficient hepatic reserve, i.e., TARE naïve normal liver in patients who had an approximately 50% higher perfused liver volume absorbed dose at the time of radioembolization [1]. For example, tolerability was similar in both the multi-compartment and single-compartment arms of DOSISPHERE-01 [1].

In this context, it is therefore not surprising that an association was not identified for NTAD. The challenge in understanding dose-toxicity relationships with TARE is due in part to these low rates of observed toxicity, as well as the fact that many providers may be “underdosing” patients relative to previously published studies [1, 17]. With this knowledge of the low rates of toxicity within the current dosing paradigm, it may be possible to increase tumor absorbed dose and better optimize treatment for patients.

Although the primary endpoint for TARGET was not evaluable due to the limited number of events, the secondary endpoints provide guidance on TAD dosimetry targets. These include associations between TAD with tumor response, AFP response, and OS. In the prospective, open-label, randomized DOSISPHERE-01 study, investigators compared clinical outcomes and safety in patients who received personalized, multi-compartment dosimetry approaches with patients who were treated using a standardized, single-compartment dosimetry approach [1]. DOSISPHERE-01 evaluated a previously identified TAD threshold of ≥ 205 Gy with a goal of 250–300 Gy when possible using multi-compartment dosimetry. DOSISPHERE-01 reported an approximate doubling of tumor response and CR by European Association for the Study of the Liver (EASL) criteria, with a more than doubling of median OS in HCC patients with a mean tumor volume exceeding 10 cm. While the mean absorbed dose to the perfused volume and mean index lesion was higher in DOSISPHERE-01 than in TARGET, the larger number of patients in TARGET allowed subgroup analysis to be conducted to further delineate the association between TAD, tumor response, and OS [1]. Much like in DOSISPHERE-01, a higher TAD in TARGET yielded an improved response rate, more than doubling CR relative to the lower TAD subgroups. Further, median OS increased from the lowest to middle to highest TAD subgroups, with the median OS of the highest subgroup (36.7 months for > 300 Gy) being more than double that of the lowest TAD subgroup (16.1 months for < 200 Gy). While there were some key differences in study population between DOSISPHERE-01 and TARGET (e.g., tumor size, Child–Pugh status, BCLC B differences), the TARGET and DOSISPHERE-01 studies collectively support the use of multi-compartment dosimetry based on ^99m^Tc-MAA pre-treatment planning. Additionally, these data provide support to the hypothesis that increasing TAD improves patient outcomes for tumor response, AFP response, and OS in a dose-dependent manner. Furthermore, these studies demonstrate multi-compartment dosimetry principles based on ^99m^Tc-MAA pre-treatment planning are likely to have broad applicability across institutions.

Limitations of the TARGET study include those often associated with retrospective, medical-record based studies, such as missing data and those associated with hospital-related processes. The retrospective nature of the study may have affected the clinical AE recording; however, Grade 3 or higher hyperbilirubinemia would not have been missed (that is, Grade 3 or higher hyperbilirubinemia was thoroughly recorded and verified at all sites). There were insufficient events to generate a dose-toxicity relationship; however, toxicity was expectedly low and helps to support the safety argument for such treatment. Peri-procedural medication varied widely and included among others, steroids and proton pump inhibitors, in different doses and regimens. Data were collected from multiple centers with heterogeneous populations and practices; while this presents some limits, it is representative of real-world data. One example is that, at many centers, PVT was included in the tumoral volume, which could have influenced results. An additional limitation relates to the collection and analysis of real-world data, e.g., patients who transitioned back to a primary care physician prior to undergoing subsequent treatment. Another limitation is that conclusions were based on pre-treatment dosimetry only, as limited post-treatment imaging availability was not sufficient for comparison. Additionally, multi-compartment dosimetry was determined retrospectively [1]. The assessment of tumor response was done at each individual institution, rather than by a central review group, potentially introducing some variation in interpretation by site. There was some heterogeneity in the type of imaging that was used; specifically, a small subset of patients had SPECT rather than SPECT/CT imaging on which their dosimetric calculations were based.

The aim of at least 205 Gy for TAD, as was proposed in recommendations on ^90^Y glass microspheres in HCC and validated in DOSISPHERE-01, was confirmed by our data with a median TAD of 225.5 Gy for responders (95% CI = 201.0–253.0 Gy) compared with 188.3 Gy for non-responders (95% CI = 164.6–215.3 Gy), especially looking at the lower and upper 95% CI level of responders and non-responders respectively [1, 3]. Increasing TAD has been shown to lead to increased objective tumor response and overall survival in both TARGET and DOSISPHERE-01. [1]

In conclusion, the TARGET study provides real-world data confirming a significant association between TAD and objective response and between TAD and OS in HCC patients treated with ^90^Y glass microspheres. Further, increasing TAD resulted in increased probability of tumor response and longer OS without a significant increase in toxicity. The lack of relationship between occurrence of grade 3 or higher hyperbilirubinemia and NTAD suggests that there is further room for dose escalation and optimization specifically in HCC, in which the tumors are hypervascular, thus enabling higher absorbed dose within the tumor while maintaining the normal liver parenchyma absorbed dose within acceptable limits.

## Supplementary Information

Below is the link to the electronic supplementary material.

Supplementary file1 (PDF 245 KB)

## Abbreviations

HCC: Hepatocellular carcinoma
TARE: Transarterial radioembolization
Y-90: Yttrium-90
TAD: Tumor absorbed dose
NTAD: Normal tissue absorbed dose
^99m^Tc-MAA: Technetium-99 m macroaggregated albumin
SPECT/CT: Single photon emission computed tomography/computed tomography
PET/CT: Positron emission tomography/computed tomography
MIRD: Medical internal radiation dose
PVT: Portal vein thrombosis
AHC: Atrophy-hypertrophy complex
TARGET: The TheraSphere™ Advanced Dosimetry Retrospective Global Study Evaluation in Hepatocellular Carcinoma Treatment
AE: Adverse event
SAE: Serious adverse event
OS: Overall survival
RECIST: Response Evaluation Criteria in Solid Tumors
TACE: Transarterial chemoembolization
BCLC: Barcelona Clinic Liver Cancer
ECOG: Eastern Cooperative Oncology Group
CR: Complete response
PR: Partial response
AFP: Alpha fetoprotein
CTCAE: Common Terminology Criteria for Adverse Events
MRI: Magnetic resonance imaging
VOI: Volume of interest
ORR: Objective response rate
mRECIST: Modified Response Evaluation Criteria in Solid Tumors
ALBI: Albumin-Bilirubin
EASL: European Association for the Study of the Liver
