# Supplementary material for: A global evaluation of advanced dosimetry in transarterial radioembolization of hepatocellular carcinoma with Yttrium-90: the TARGET study
Source: Eur J Nucl Med Mol Imaging. 2022 Apr 8;49(10):3340–52. doi: 10.1007/s00259-022-05774-0 (PMC9308596; doi:10.1007/s00259-022-05774-0)
Supplement: Supplementary file 1 — Supplementary file1 (PDF 245 KB) [file 259_2022_5774_MOESM1_ESM.pdf]

Supplementary Information

A Global Evaluation of Advanced Dosimetry in Transarterial Radioembolization of Hepatocellular Carcinoma with Yttrium-90: The TARGET Study

Marnix Lam, MD, PhD[1]; Etienne Garin, MD, PhD[2]; Marco Maccauro, MD[3]; S. Cheenu Kappadath, PhD[4]; Daniel Y. Sze, MD, PhD[5]; Cuneyt Turkmen, MD[6]; Murat Cantasdemir, MD[7]; Paul Haste, MD[8]; Ken Herrmann, MD[9]; Hamad Saleh Alsuhaibani, MD[10]; Matthew Dreher, PhD[11]; Kirk D. Fowers, PhD[11]; Riad Salem, MD[12]

1. Department of Radiology and Nuclear Medicine, University Medical Center Utrecht, The Netherlands
2. Nuclear Medicine Department, Eugene Marquis Center, Rennes, France
3. Nuclear Medicine Unit, Fondazione IRCCS Istituto Nazionale dei Tumori, Italy
4. Department of Imaging Physics, University of Texas MD Anderson Cancer Center, Houston, TX, USA
5. Division of Interventional Radiology, Stanford University, Stanford, CA, USA
6. Department of Nuclear Medicine, Istanbul Faculty of Medicine, Istanbul University, Istanbul, Turkey
7. Division of Interventional Radiology, Istanbul Florence Nightingale Hospital, Istanbul, Turkey
8. Department of Clinical Radiology and Imaging Sciences, Indiana University School of Medicine, Indianapolis, IN, USA
9. Department of Nuclear Medicine, University of Duisburg-Essen, and German Cancer Consortium (DKTK)-University Hospital Essen, Essen, Germany
10. Department of Radiology, King Faisal Specialist Hospital, Riyadh, Saudi Arabia
11. Boston Scientific Corporation, Marlborough, MA, USA
12. Department of Radiology, Northwestern Feinberg School of Medicine, Chicago, IL, USA

Corresponding Author:

Marnix Lam: m.lam@umcutrecht.nl

<u>Table of Contents</u>

Table S1. Univariable and Multivariable Logistic Regression Analyses of Objective Response by mRECIST

Table S2. Univariable and Multivariable Logistic Regression Analyses of Objective Response by RECIST 1.1

Table S3. Univariable and Multivariable Cox Regression Analyses of Overall Survival

Table S1. Univariable and Multivariable Logistic Regression Analyses of Objective Response by mRECIST

Variable	Category	Univariable Analyses		Final Multivariable Analysis	
		Odds Ratio (95% CI)	P-value	Odds Ratio (95% CI)	P-value
Tumor Absorbed Dose[a]				1.26 (1.02 – 1.55)	0.033
Gender	Male	0.47 (0.22 – 1.01)	0.052	0.43 (0.19 – 0.94)	0.035
	Female	1		1	
Ascites at Baseline	Slight	0.78 (0.32 – 1.92)	0.589	--	--
	Absent	1		--	
Splenomegaly at Baseline	Present	0.59 (0.33 – 1.04)	0.067	--	--
	Absent	1		--	
HCC Liver Status	Liver Cirrhosis	0.95 (0.32 – 2.82)	0.916	--	--
	Liver Fibrosis	1.36 (0.19 – 9.75)		--	
	Normal Liver	1		--	
HCC Etiology	Alcohol Use	1.35 (0.49 – 3.76)	0.686	--	--
	Hepatitis C	1.92 (0.74 – 4.98)		--	
	NASH/NAFLD	2.39 (0.70 – 8.17)		--	
	More than one HCC Etiology	1.79 (0.51 – 6.31)		--	
	Unknown	1.32 (0.50 – 3.49)		--	
	Hepatitis B	1		--	
Largest Tumor Size at Baseline by mRECIST	< 3 cm	0.64 (0.04 – 10.76)	0.901	--	--
	≥3 to <5 cm	0.76 (0.35 – 1.64)		--	
	≥5 to <8 cm	0.95 (0.48 – 1.87)		--	
	≥ 8 cm	1		--	
Log Number of Tumors at Baseline[b]		1.05 (0.53 – 2.09)	0.880	--	--

Variable	Category	Univariable Analyses		Final Multivariable Analysis	
		Odds Ratio (95% CI)	P-value	Odds Ratio (95% CI)	P-value
Unilobar or Bilobar Disease	Bilobar	1.05 (0.56 – 1.95)	0.884	--	--
	Unilobar	1		--	
Child-Pugh at Baseline	A5	1.15 (0.65 – 2.03)	0.634	--	--
	A6 or B7	1		--	
PVT at Baseline	Present	0.86 (0.47 – 1.57)	0.628	--	--
	Absent	1		--	
Untreated liver fraction[c]		0.99 (0.97 – 1.01)	0.272	--	--
Prior Sorafenib Treatment	Yes	0.96 (0.38 – 2.43)	0.931	--	--
	No	1			
BCLC Stage at Baseline	A	0.58 (0.24 – 1.42)	0.483	--	--
	B	0.94 (0.50 – 1.77)		--	
	C	1		--	
ALBI Score at Baseline	1	1.48 (0.81 – 2.70)	0.198	--	--
	2 or 3	1		--	
PVT ^{99m}Tc-MAA targeting	No PVT	0.99 (0.37 – 2.62)	0.889	--	--
	No PVT targeting	1.15 (0.56 – 2.40)		--	
	PVT targeting	1		--	
CT or MRI for Diagnostic and Response Imaging	CT	1.65 (0.83 – 3.31)	0.156	--	--
	MRI	1		--	
Pre-procedural ^{99m}Tc-MAA SPECT vs SPECT/CT	^{99m}Tc-MAA SPECT	0.69 (0.32 – 1.50)	0.347	--	--
	SPECT/CT	1		--	
Week of TARE Administration	Second	1.46 (0.79 – 2.70)	0.231	--	--
	First	1		--	
Deformable Registration	Yes	1.54 (0.85 – 2.81)	0.158	--	--
	No	1		--	

Variable	Category	Univariable Analyses		Final Multivariable Analysis	
		**Odds Ratio (95% CI)**	**P-value**	**Odds Ratio (95% CI)**	**P-value**
Similarity Score Between Injection Locations[d]	1	0.60 (0.28 – 1.28)	0.359	--	--
	2	0.53 (0.19 – 1.48)		--	
	3	1		--	
Bilirubin at Baseline	<1.0 mg/dL	3.22 (1.74 – 5.95)	<0.001	3.39 (1.81 – 6.34)	<0.001
	≥1.0 mg/dL	1		1	
Albumin at Baseline	<3.6 g/dL	0.96 (0.54 – 1.72)	0.888	--	--
	≥3.6 g/dL	1		--	

CI = Confidence Interval

[a] Odds ratio for tumor absorbed dose corresponds to a 100 Gy increase in absorbed dose.

[b] Odds ratio corresponds to a 1 unit increase in this variable where 69.4% of patients had a solitary tumor, 21.5% had two tumors and 9.1% had ≥3 tumors.

[c] Odds ratio corresponds to a 1 unit increase in this variable where 33% of patients had an untreated liver fraction of <30%, 46.9% were 30-50% and 20.1% were >50%.

[d] Odds ratio corresponds to comparison of similarity scores between injection locations for ^{99m}Tc-MAA and TARE of 1) nearly identical (≤1 cm) with no influence of bifurcations, 2) similar location (>1 and ≤2 cm) with no influence of bifurcations,3) dissimilar location (>2 cm) or influence of a bifurcation.

Table S2. Univariable and Multivariable Logistic Regression Analyses of Objective Response by RECIST 1.1

Variable	Category	Univariable Analyses		Final Multivariable Analysis	
		Odds Ratio (95% CI)	P-value	Odds Ratio (95% CI)	P-value
Tumor Absorbed Dose[a]				1.22 (1.02 – 1.47)	0.028
Gender	Male	0.66 (0.33 – 1.33)	0.250	--	--
	Female	1		--	
Ascites at Baseline	Slight	0.76 (0.28 – 2.04)	0.581	--	--
	Absent	1		--	
Splenomegaly at Baseline	Present	0.91 (0.51 – 1.62)	0.741	--	--
	Absent	1		--	
HCC Liver Status	Liver Cirrhosis	2.03 (0.55 – 7.53)	0.569	--	--
	Liver Fibrosis	0.00 (0.00 – >999)		--	
	Normal Liver	1		--	
HCC Etiology	Alcohol Use	1.74 (0.60 – 5.07)	0.363	--	--
	Hepatitis C	1.35 (0.50 –3.67)		--	
	NASH/NAFLD	0.58 (0.15 – 2.16)		--	
	More than one HCC Etiology	1.24 (0.34 – 4.45)		--	
	Unknown	0.74 (0.25 – 2.17)		--	
	Hepatitis B	1		--	
Largest Tumor Size at Baseline by mRECIST	< 3 cm	2.50 (0.15 – 41.96)	0.880	--	--
	≥3 to <5 cm	1.07 (0.48 – 2.35)		--	
	≥5 to <8 cm	1.22 (0.61 – 2.42)		--	
	≥ 8 cm	1		--	
Log Number of Tumors at Baseline[b]		1.51 (0.77 – 2.98)	0.231	--	--
Unilobar or Bilobar Disease	Bilobar	1.12 (0.59 – 2.10)	0.731	--	--

Variable	Category	Univariable Analyses		Final Multivariable Analysis	
		Odds Ratio (95% CI)	P-value	Odds Ratio (95% CI)	P-value
	Unilobar	1		--	
Child-Pugh at Baseline	A5	0.97 (0.54 – 1.74)	0.909	--	--
	A6 or B7	1		--	
PVT at Baseline	Present	1.14 (0.61 – 2.12)	0.675	--	--
	Absent	1		--	
Untreated liver fraction[c]		0.99 (0.97 – 1.00)	0.126	--	--
Prior Sorafenib Treatment	Yes	1.45 (0.56 – 3.76)	0.443	--	--
	No	1		--	
BCLC Stage at Baseline	A	0.45 (0.16 – 1.27)	0.306	--	--
	B	0.97 (0.51 – 1.84)		--	
	C	1		--	
ALBI Score at Baseline	1	1.11 (0.61 – 2.01)	0.743	--	--
	2 or 3	1		--	
PVT ^{99m}Tc-MAA targeting	No PVT	0.97 (0.35 – 2.67)	0.914	--	--
	No PVT targeting	0.87 (0.41 – 1.83)		--	
	PVT targeting	1		--	
CT or MRI for Diagnostic and Response Imaging	CT	1.25 (0.60 – 2.63)	0.549	--	--
	MRI	1		--	
Pre-procedural ^{99m}Tc- MAA SPECT vs SPECT/CT	^{99m}Tc-MAA SPECT	0.87 (0.38 – 1.98)	0.738	--	--
	SPECT/CT	1		--	
Week of TARE Administration	Second	1.52 (0.83 – 2.81)	0.177	--	--
	First	1		--	
Deformable Registration	Yes	1.50 (0.83 – 2.71)	0.184	--	--
	No	1		--	
Similarity Score Between Injection Locations[d]	1	1.20 (0.57 – 2.56)	0.647	--	--

Variable	Category	Univariable Analyses		Final Multivariable Analysis	
		**Odds Ratio (95% CI)**	**P-value**	**Odds Ratio (95% CI)**	**P-value**
	2	1.65 (0.58 – 4.70)		--	
	3	1		--	
Bilirubin at Baseline	<1.0 mg/dL	2.38 (1.21 – 4.68)	0.012	2.38 (1.21 – 4.68)	0.012
	≥1.0 mg/dL	1		1	
Albumin at Baseline	<3.6 g/dL	1.18 (0.65 – 2.15)	0.581	--	--
	≥3.6 g/dL	1		--	

CI = Confidence Interval

[a] Odds ratio for tumor absorbed dose corresponds to a 100 Gy increase in absorbed dose.

[b] Odds ratio corresponds to a 1 unit increase in this variable where 69.4% of patients had a solitary tumor, 21.5% had two tumors and 9.1% had ≥3 tumors.

[c] Odds ratio corresponds to a 1 unit increase in this variable where 33% of patients had an untreated liver fraction of <30%, 46.9% were 30-50% and 20.1% were >50%.

[d] Odds ratio corresponds to comparison of similarity scores between injection locations for ^{99m}Tc-MAA and TARE of 1) nearly identical (≤1 cm) with no influence of bifurcations, 2) similar location (>1 and ≤2 cm) with no influence of bifurcations,3) dissimilar location (>2 cm) or influence of a bifurcation.

Table S3. Univariable and Multivariable Cox Regression Analyses of Overall Survival

Variable	Category	Univariable Analyses		Final Multivariable Analysis	
		Hazard Ratio (95% CI)	P-value	Hazard Ratio (95% CI)	P-value
Tumor Absorbed Dose[a]				0.84 (0.72– 0.97)	0.016
Gender	Male	1.27 (0.77– 2.11)	0.354	--	--
	Female	1		--	
Ascites at Baseline	Slight	2.33 (1.33– 4.08)	0.003	--	--
	Absent	1		--	
Splenomegaly at Baseline	Present	1.44 (0.97 – 2.13)	0.068	--	--
	Absent	1		--	
HCC Liver Status	Liver Cirrhosis	1.29 (0.56 – 2.97)	0.783	--	--
	Liver Fibrosis	0.99 (0.20 – 4.94)		--	
	Normal Liver	1		--	
HCC Etiology	Alcohol Use	1.02 (0.50 – 2.10)	0.982	--	--
	Hepatitis C	0.89 (0.44 – 1.79)		--	
	NASH/NAFLD	1.17 (0.52 – 2.60)		--	
	More than one HCC Etiology	1.10 (0.44 – 2.73)		--	
	Unknown	1.02 (0.51 – 2.03)		--	
	Hepatitis B	1		--	
Largest Tumor Size at Baseline by mRECIST	< 3 cm	0.45 (0.06 – 3.33)	0.879	--	--
	≥3 to <5 cm	1.01 (0.62 – 1.65)		--	
	≥5 to <8 cm	0.95 (0.60 – 1.50)		--	
	≥ 8 cm	1		--	
Log Number of Tumors at Baseline[b]		0.79 (0.48 – 1.30)	0.344	--	--
Unilobar or Bilobar Disease	Bilobar	1.47 (0.98 – 2.22)	0.066	1.55 (1.02 – 2.34)	0.041
	Unilobar	1		1	

Variable	Category	Univariable Analyses		Final Multivariable Analysis	
		Hazard Ratio (95% CI)	P-value	Hazard Ratio (95% CI)	P-value
Child-Pugh at Baseline	A5	0.65 (0.44 – 0.96)	0.030	--	--
	A6 or B7	1		--	
PVT at Baseline	Present	2.64 (1.77 – 3.92)	<0.0001	2.61 (1.75 – 3.89)	<0.0001
	Absent	1		1	
Untreated liver fraction[c]		1.01 (1.00 – 1.02)	0.129	--	--
Prior Sorafenib Treatment	Yes	1.15 (0.61 – 2.17)	0.674	--	--
	No	1		--	
BCLC Stage at Baseline	A	0.36 (0.17 – 0.79)	0.020	--	--
	B	0.70 (0.46 – 1.06)		--	
	C	1		--	
ALBI Score at Baseline	1	0.66 (0.44 – 0.99)	0.046	--	--
	2 or 3	1		--	
PVT ^{99m}Tc-MAA targeting	No PVT	0.46 (0.28 – 0.73)	<0.0001	--	--
	No PVT targeting	1.64 (0.90 – 2.98)		--	
	PVT targeting	1		--	
CT or MRI for Diagnostic and Response Imaging[d]	CT	0.61 (0.39 – 0.95)	0.028	0.58 (0.37 – 0.91)	0.017
	MRI	1		1	
Pre-procedural ^{99m}Tc- MAA SPECT vs SPECT/CT	^{99m}Tc-MAA SPECT	1.37 (0.84 – 2.23)	0.214	--	--
	SPECT/CT	1		--	
Week of TARE Administration	Second	1.13 (0.74 – 1.72)	0.583	--	--
	First	1		--	
Deformable Registration	Yes	0.92 (0.61 – 1.38)	0.677	--	--
	No	1		--	
Similarity Score Between Injection Locations[e]	1	0.85 (0.52 – 1.39)	0.760	--	--

Variable	Category	Univariable Analyses		Final Multivariable Analysis	
		Hazard Ratio (95% CI)	P-value	Hazard Ratio (95% CI)	P-value
	2	0.99 (0.52 – 1.86)		--	
	3	1		--	
Bilirubin at Baseline	<1.0 mg/dL	0.76 (0.51 – 1.14)	0.184	--	--
	≥1.0 mg/dL	1		--	
Albumin at Baseline	<3.6 g/dL	1.31 (0.89 – 1.94)	0.175	--	--
	≥3.6 g/dL	1		--	

CI = Confidence Interval

[a] Hazard ratio for tumor absorbed dose corresponds to a 100 Gy increase in absorbed dose.

[b] Hazard ratio corresponds to a 1 unit increase in this variable where 69.4% of patients had a solitary tumor, 21.5% had two tumors and 9.1% had ≥3 tumors.

[c] Hazard ratio corresponds to a 1 unit increase in this variable where 33% of patients had an untreated liver fraction of <30%, 46.9% were 30-50% and 20.1% were >50%.

[d] The impact on OS due to CT versus MRI for diagnostic and response imaging described above is unexpected and is likely due to confounding with other clinical factors that were not recorded in the study and so could not be included in the modelling performed, and so should be interpreted with caution.

[e] Hazard ratio corresponds to comparison of similarity scores between injection locations for ^{99m}Tc-MAA and TARE of 1) nearly identical (≤1 cm) with no influence of bifurcations, 2) similar location (>1 and ≤2 cm) with no influence of bifurcations,3) dissimilar location (>2 cm) or influence of a bifurcation.
